# Culture Change and Quality Star Ratings in High Medicaid Nursing Homes: Does Time of Adoption Make a Difference?

**DOI:** 10.1093/geroni/igaa057.2362

**Published:** 2020-12-16

**Authors:** Latarsha Chisholm, Akbar Ghiasi, Justin Lord, Robert Weech-Maldonado

**Affiliations:** 1 University of Central Florida, Orlando, Florida, United States; 2 University of Incarnate Word, San Antonio, Texas, United States; 3 LSU-Shreveport, Shreveport, Louisiana, United States; 4 UAB, Birmingham, Alabama, United States

## Abstract

Racial/ethnic disparities have been well documented in long-term care literature. Culture change is a movement to transition nursing homes to more home-like environments to improve the quality of care for all residents. The purpose of this study was to examine how the involvement of culture change initiatives among high Medicaid facilities was associated with nursing home quality. The study relied on both survey and secondary nursing home data for the years 2017-2018. The sample included high Medicaid facilities. The final model consisted of an ordinal logistic regression. High-Medicaid nursing homes with six or more years in culture change initiatives had higher odds of having a higher star rating, while facilities with one year or less had significantly lower odds of having a higher star rating. Culture change initiatives may require some time to effectively implement, but these initiatives are potential mechanisms to improve quality in high Medicaid nursing homes.

